# Waist–hip ratio measured by bioelectrical impedance analysis as a valuable predictor of chronic kidney disease development

**DOI:** 10.1186/s12882-022-02981-7

**Published:** 2022-11-01

**Authors:** Younghoon Song, Jeong Ah Hwang, Jaeun Shin, Eunjung Cho, Shin Young Ahn, Gang Jee Ko, Young Joo Kwon, Ji Eun Kim

**Affiliations:** 1 Department of Internal Medicine, Korea University Guro Hospital, Gurodong-Ro 148, Guro-Gu, Seoul, South Korea; 2 Department of Internal Medicine, Korea University College of Medicine, Seoul, South Korea

**Keywords:** Bioelectrical impedance analysis, Body mass index, Chronic kidney disease, Obesity

## Abstract

Obesity is a major health problem worldwide and is associated with chronic kidney disease (CKD). Body mass index (BMI) is a common method of diagnosing obesity, but there are concerns about its accuracy and ability to measure body composition. This study evaluated the risk of CKD development in a middle-aged population in association with various body composition metrics. From a prospective cohort of 10,030 middle-aged adults, we enrolled 6727 for whom baseline and follow-up data were available. We collected data pertaining to participants' BMI, manually measured waist–hip ratio (WHR), and various measurements of bioelectrical impedance analysis (BIA), including total body fat content, muscle content, and calculated WHR, and classified the participants into quintiles accordingly. CKD was defined as an estimated glomerular filtration rate (eGFR) < 60 ml/min/1.73 m^2^ in follow-up laboratory tests. While an increase in BMI, WHR, and total body fat were associated with an elevated risk of CKD, an increase in total body muscle decreased the risk. Among the body composition metrics, WHR measured by BIA had the highest predictive value for CKD (C-statistics: 0.615). In addition, participants who were “healthy overweight, (defined as low WHR but high BMI), exhibited a 62% lower risk of developing CKD compared to those with “normal-weight obesity,” (defined as high WHR despite a normal BMI). In conclusion, we suggest that central obesity measured by BIA is a more accurate indicator than BMI for predicting the development of CKD.

## Introduction

Over the past few decades, the number of overweight and obese adults has increased sequentially and rapidly [1, 2]. Prevention and treatment of obesity as a type of “metabolic disorder” has become an important objective of public health care worldwide [2–4]. According to the World Health Organization definitions, the terms overweight and obesity are defined as abnormal fat accumulation, which is commonly defined and classified using the body mass index, which is an easily measurable metric [5, 6]. However, even though body mass index (BMI) can easily calculated using body weight and height, it cannot reflect body fat distribution or distinguish the ratio of non-fat and fat contents [7, 8]. As a substitute for BMI, other indicators of obesity, including waist circumference, hip circumference, waist–hip ratio (WHR), and fat volume measurement by imaging tools or bioelectrical impedance analysis (BIA), can be used to measure fat content and define visceral obesity [7, 9].

Obesity has been shown to be associated with several kidney diseases in previous studies [10–18]. A higher BMI is related to the development of proteinuria, decreased eGFR, and an increased incidence of end stage renal disease (ESRD) [10–12]. Furthermore, a higher BMI is associated with the development of nephrolithiasis as well as kidney cancer [13–16]. Despite the close relationship between kidney disease and BMI, the association between kidney dysfunction and fat measurement metrics other than BMI have not been well studied [19, 20]. Moreover, no study has compared the predictability of kidney dysfunction according to obesity calculation metrics.

In this study, we evaluated the risk of developing kidney dysfunction in a middle-aged population in association with various body composition metrics. In addition, we aimed to identify the most significant obesity-related factors associated with kidney dysfunction to improve our understanding of the link between obesity and kidney disease.

## Methods

### Study setting and cohort

This observational study using a community-based prospective cohort was approved by the Institutional Review Board of Korea University Guro Hospital (IRB No. 2021GR0573) and was conducted in compliance with the principles of the Declaration of Helsinki. Informed consent was waived by the Institutional Review Board of Korea University Guro Hospital because of the public data usage. For this analysis, we assessed patients from the Ansan and Anseong population-based cohort from the Korean Genome and Epidemiology Study (KoGES). The KoGES cohort consisted of 10,030 middle-aged adults living in Ansan (urban area) and Anseong (rural area) in Gyeonggi-do, South Korea. The design of the cohort was previously published [21]. Of the patients in the KoGES cohort, we excluded those with eGFR < 60 ml/min/1.73 m^2^ or kidney disease at baseline, no follow-up eGFR data, or no BIA data.

### Data collection and definition

The KoGES cohort was evaluated for anthropometric data, lifestyle patterns, medical history, and laboratory results at baseline and followed up every 2 years. For this study, a database including data from the baseline survey in 2001 to the 7^th^ follow-up survey was used.

We collected demographic and anthropometric data, including that pertaining to age, sex, weight, height, waist circumference, and hip circumference. Lifestyle patterns, including alcohol consumption and smoking habits, were also recorded. History of hypertension and diabetes mellitus and laboratory results, including albumin and creatinine levels, were collected. The eGFR was calculated using the CKD Epidemiology Collaboration creatinine equation [22].

For body composite analysis, all participants underwent BIA using InBody 3.0; Biospace, Seoul, Korea [23, 24]. The principal of BIA is that electric current passes through the body at a differential rate depending on body composition [25]. Based on the electrical impedance of tissue water, the device estimates the amount of body fat, muscle, bone and water; and calculates the WHR based on the data obtained [26]. Skeletal muscle ratio (%) calculated by skeletal muscle mass divided by body weight, total body fat ratio (%) calculated by body fat mass divided by body weight, and waist–hip ratio were obtained from BIA. BMI was calculated as body weight (kg) divided by the square of the height (m). The manually measured waist–hip ratio was calculated as waist circumference divided by hip circumference, and each circumferences was calculated as the mean value of three measurements.

### Study outcome

The main study outcome was the development of CKD. CKD development was defined as eGFR < 60 ml/min/1.73 m^2^ at any time during follow-up. Since the date of measurement information was only available up to the month, censoring date was regarded as the first day of the month.

### Statistical analysis

Baseline variables are shown as means with standard deviations for continuous variables and numbers and percentages for categorical variables. Univariate and multivariate Cox regression analyses were performed to measure the risk of CKD development. The Kaplan–Meier curve was used to visualize the risks over time. Receiver operating characteristic curve analyses (ROC) were used to predict CKD development, and the area under the ROC curve of each variable was compared using permutation tests. A *P*-value of < 0.05 on two-sided tests was set to indicate statistical significance. All analyses were performed using Stata version 15.0.

## Results

### Baseline characteristics

**Fig. 1:**
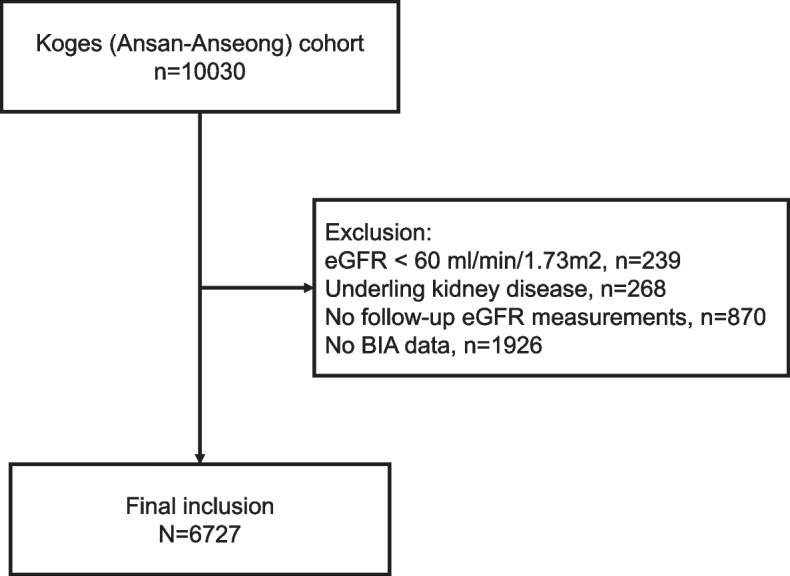
Flow chart of the enrollment of participants

We excluded 3303 from the cohort (total, 10,030) and included the remaining 6727 in this study (Fig. 1). The median follow-up duration was 5264 (3075–5417) days. Among the study participants, 1609 (23.9%) developed CKD during a mean follow-up period of 3530 (1400–4260) days. The mean age of participants was 51.3 ± 8.7 years, and 48.9% were men. The baseline eGFR was 92.1 ± 12.9 ml/min/1.73 m^2^. The participants had a mean BMI of 24.6 ± 3.1 kg/m^2^. In body composition analysis, the WHR calculated by BIA was 0.90 ± 0.05 and the WHR calculated by manually measured circumferences was 0.87 ± 0.08. The baseline characteristics of the participants are presented in Table 1.

**Table 1:** Baseline characteristics of the participants

Variables	*n* = 6727
Age, year	51.3 ± 8.7
Male sex, n(%)	3286 (48.9)
HTN, n(%)	899 (13.4)
DM, n(%)	413 (6.1)
Alcohol consumption, n(%)	
Never	2997 (44.9)
Ex-drinker	405 (6.1)
Current	3269 (49)
Smoking, n(%)	
Never	3891 (58.5)
Ex-smoker	1089 (16.4)
Current	1677 (25.2)
BMI, kg/m^2^	24.6 ± 3.1
WHR (manual)	0.87 ± 0.08
WHR (BIA)	0.90 ± 0.05
Total fat ratio, %	26.8 ± 7.1
Skeletal muscle ratio, %	69.1 ± 6.8
eGFR, ml/min/1.73m^2^	92.1 ± 12.9
Serum albumin, g/dL	4.3 ± 0.3

Abbreviations: *HTN* hypertension, *DM* diabetes mellitus, *BMI* body mass index, *WHR* waist-hip ratio, *BIA* bioelectrical impedence analysis, *eGFR* estimated glomerular filtration rate

### Association between development of CKD and body composition metrics

**Fig. 2:**
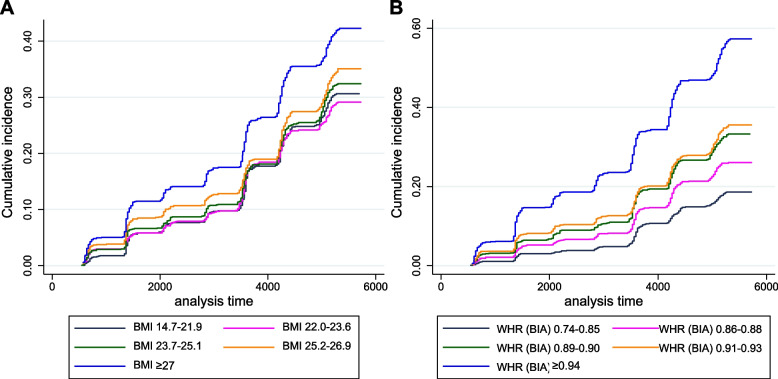
Cumulative incidence of chronic kidney disease (CKD) according to quintiles of BMI (left) and WHR measured by BIA (right). BMI, body mass index; WHR, waist hip ratio; BIA, bioelectrical impedance analysis

To compare the hazard ratios for CKD development, we divided each composite metric into quintile groups (Table 2). As a result, an increase in BMI, the traditional body composite metric, showed a significant positive association with elevated CKD risk. The participants who had a BMI > 27 kg/m^2^ showed 1.28 times higher risk of developing CKD compared to the participants who had a BMI of 22.0 − 23.6 kg/m^2^ (Fig. 2A). On the other hand, the total body fat ratio calculated by BIA also showed an association with CKD development, but not in a sequential manner. Skeletal muscle ratio calculated by BIA showed a negative association with CKD risk, and the participants in the highest quintile of muscle mass ratio showed 0.79 times the CKD risk compared to that of the lowest quintile.

**Table 2:** The risk of CKD development according to various body composition profiles

	Univariable		Multivariable^*^	
Quintiles	HR (95% CI)	*P*-value	HR (95% CI)	*P*-value
BMI (kg/m^2^)				
14.7–21.9	1.03 (0.87–1.22)	0.701	0.98 (0.83–1.16)	0.794
22.0–23.6	1 (ref)		1 (ref)	
23.7–25.1	1.10 (0.94–1.30)	0.239	1.08 (0.92–1.28)	0.341
25.2–26.9	1.20 (1.03–1.41)	0.023	1.05 (0.90–1.24)	0.515
≥ 27	1.49 (1.28–1.74)	< 0.001	1.28 (1.09–1.50)	0.002
Total body fat ratio (%)				
7.1–20.1	1 (ref)		1 (ref)	
20.2–24.4	1.32 (1.11–1.58)	0.002	1.21 (1.01–1.46)	0.038
24.5–29.1	1.36 (1.14–1.62)	0.001	1.15 (0.96–1.40)	0.135
29.2–33.1	1.68 (1.42–1.99)	< 0.001	1.16 (0.94–1.43)	0.155
≥ 33.2	2.38 (2.02–2.79)	< 0.001	1.33 (1.07–1.65)	0.009
Skeletal musle ratio (%)				
39.7–62.8	1 (ref)		1 (ref)	
62.9–66.7	0.70 (0.61–0.81)	< 0.001	0.88 (0.76–1.01)	0.078
66.8–71.1	0.58 (0.50–0.67)	< 0.001	0.88 (0.75–1.04)	0.127
71.2–75.2	0.56 (0.48–0.64)	< 0.001	0.94 (0.78–1.14)	0.528
≥ 75.3	0.42 (0.36–0.50)	< 0.001	0.79 (0.63–0.98)	0.03
WHR (manual)				
0.63–0.80	1 (ref)		1 (ref)	
0.81–0.85	1.12 (0.93–1.34)	0.223	0.84 (0.70–1.02)	0.075
0.86–0.89	1.39 (1.17–1.65)	< 0.001	0.91 (0.76–1.09)	0.313
0.90–0.93	1.59 (1.34–1.89)	< 0.001	0.90 (0.75–1.08)	0.27
≥ 0.94	2.31 (1.97–2.70)	< 0.001	0.94 (0.79–1.11)	0.455
WHR (BIA)				
0.74–0.85	1 (ref)		1 (ref)	
0.86–0.88	1.42 (1.17–1.72)	< 0.001	1.09 (0.90–1.33)	0.387
0.89–0.90	1.82 (1.50–2.20)	< 0.001	1.13 (0.92–1.37)	0.244
0.91–0.93	1.95 (1.62–2.34)	< 0.001	1.07 (0.89–1.30)	0.47
≥ 0.94	3.23 (2.71–3.85)	< 0.001	1.24 (1.02–1.50)	0.027

Abbreviations: *BMI* body mass index, *WHR* waist-hip ratio, *BIA* bioelectrical impedance analysis, *HR* hazard ratio, *CI* confidence interval

^*^Multivariable analysis was adjusted with age, sex, alcohol consumption, smoking status, serum albumin, estimated glomerular filtration rate, hypertension and diabetes

When comparing manually measured and BIA-calculated WHRs, the quintile ranges of each metric were different from each other. The association for CKD risk in the quintile groups of manually measured WHR was not significant, while the BIA-calculated WHR was significantly associated with CKD, especially in the highest quintile. A WHR level of 0.94 or higher was the highest quintile group in both metrics, but the risk of CKD increased by 1.24 times only in the BIA-calculated WHR group compared with the 1st quintile group (Fig. 2B).

### Comparison of predictive power for CKD for each body composition metric

**Fig. 3:**
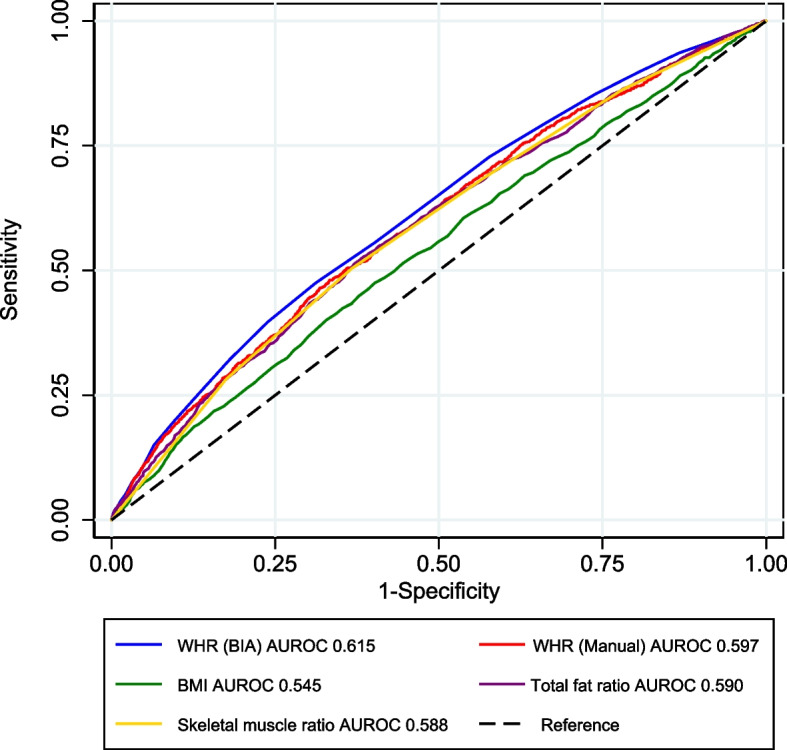
Receiver operating characteristic (ROC) analysis for chronic kidney disease (CKD) development according to various body composition profiles. The numbers in the legend represent areas under the ROC curve (C-statistics). WHR, waist–hip ratio; BIA, bioelectrical impedance analysis; BMI, body mass index; AUROC, area under receiver operating characteristic curve

Receiver operating curve (ROC) analysis was performed to find the most significant predictor of CKD development in body composite metrics including BMI, total body fat, manually measured WHR, and BIA-calculated WHR. Of the metrics, the area under the ROC of BIA-calculated WHR was the highest at 0.615, which was statistically higher than the area under the ROC for BMI, total body fat ratio, or skeletal muscle ratio (Fig. 3). The area under the ROC for manually measured WHR was not statistically different from that of BIA-calculated WHR (p = 0.106).

### CKD risk comparison between “normal-weight obesity” and “healthy overweight” groups

**Fig. 4:**
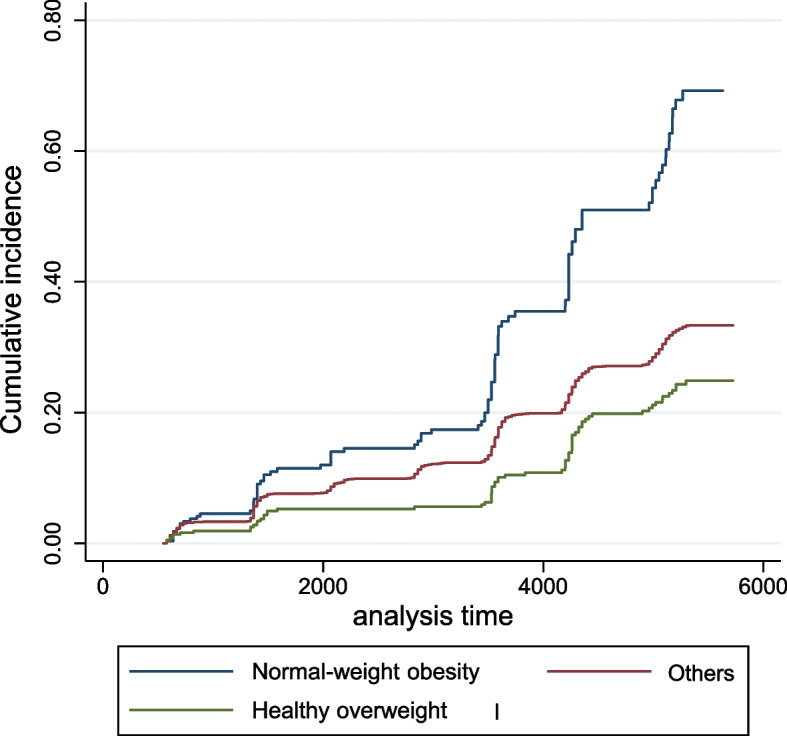
Comparison of the cumulative incidence of chronic kidney disease (CKD) in the “healthy overweight” and “normal-weight obesity” groups. The blue line represents “normal-weight obesity,” defined as a high WHR with a normal BMI. The green line represents “healthy overweight” defined as a low WHR with a high BMI. The red line represents other participants not included in the “healthy overweight” or “normal-weight obesity” categories. WHR, waist–hip ratio; BIA, bioelectrical impedance analysis; BMI, body mass index

After we found BIA-calculated WHR as the most significant predictor of CKD among body composite variables, we further evaluated the concept of “normal-weight obesity” versus “healthy overweight” on CKD development. “Normal-weight obesity” was defined as BMI < 23 kg/m^2^ in a patient with BIA-calculated WHR > 0.9; “healthy overweight” was defined as BMI > 25 kg/m^2^ in a patient with BIA-calculated WHR < 0.9. Using a Cox regression analysis, we found that “healthy overweight” individuals showed a significantly lower risk of CKD compared to “normal-weight obesity” individuals (HR 0.38, 95% CI, 0.28–0.51, *p* < 0.001; Fig. 4).

## Discussion

We observed the development of kidney dysfunction in a middle-aged population for approximately 14.4 years, and we assessed the association of various body composition metrics on kidney dysfunction in this population. While an increase in BMI, WHR, and total body fat were associated with an elevated risk of kidney dysfunction, an increase in total body muscle decreased the risk of kidney dysfunction. Among the body composition metrics, WHR measured by BIA had the highest predictive value for kidney dysfunction. In addition, when comparing the kidney dysfunction risk between the “normal-weight obesity” and “healthy overweight” categories, “healthy overweight” showed a 62% risk reduction compared to “normal-weight obesity,” suggesting the significance of a higher WHR rather than a higher BMI.

Numerous observational studies have shown an association between obesity and chronic kidney disease [10–12]. However, most studies used the traditional obesity model based on BMI, and only a few studies examined abdominal obesity using WHR or waist circumference (WC). Previous studies with WHR or WC had several limitations, such as cross-sectional design [19, 27], short duration of follow-up, and a low incidence of the outcome [20]. Although BMI is an easily measurable metric, it has a critical limitation in that it cannot assess fat distribution or muscle content in specific body areas. Several recent studies have revealed that WHR and WC correlated more with the outcomes of obesity, including diabetes and mortality, compared to BMI [28, 29]. Lee et al. assessed correlation between eGFR and waist circumference-related obesity metrics using cross-sectional data [30]. However, because of the study design, causality between obesity metrics and eGFR can not be suggested. Kjaergaard et al. used Mendelian Randomization method to estimate direct causal effect of BMI and WHR on kidney function [31]. Kjaergaard et al. focused on the verification of causality between specific body composite factor and renal dysfunction, while our study conducted for finding best predictor among body composite metrics of kidney dysfunction. The study of Hong et al. assessed multivariable regression analysis for moderate CKD to find an adequate predictive model using various obesity-related factors [32]. However, the follow up duration for renal dysfunction was relatively short (3 months), and only data on the risk ratio were presented without predictability for each variable.

The exact mechanisms by which obesity may contribute to the development or progression of CKD remain unclear. The key physiological responses of the kidney to obesity are an increase in glomerular filtration rate, renal plasma flow, filtration fraction, and tubular absorption of sodium [33, 34]. Glomerular hyperfiltration induced by obesity increases sodium delivery to the renal proximal tubule, resulting in the activation of sodium transporters in the nephron [33, 34]. As a result of intraglomerular hypertension, mechanical stress on the capillary wall increases, leading to podocyte injury and glomerulosclerosis [34]. Furthermore, the renin–angiotensin–aldosterone system and renal sympathetic nervous system are activated in obesity, and these factors contribute to the pathogenesis of obesity-related sodium retention and glomerular hyperfiltration [35–38]. Moreover, several comorbid conditions related to obesity, including hypertension and glucose intolerance, may result in deleterious renal consequences [39, 40].

However, most obese individuals do not develop CKD, and there is a high proportion of metabolically healthy obese individuals [41, 42]. Thus, increased weight alone cannot be the only factor that induces kidney damage. Regarding the results of the present study, the “healthy overweight” population, who has relatively lower visceral fat with high body weight, showed a lower risk of CKD development, suggesting a critical role of central obesity on the health and function of the kidneys. Consistently, previous studies have shown paradoxical results of obesity (defined by high BMI) on lower mortality in advanced CKD and ESRD, suggesting the ineffectiveness of using BMI as a measure of obesity [43, 44]. Adipokines such as leptin, adiponectin, resistin, and visfatin, which are mainly secreted by visceral fat, may be the cause for the specific effects of central adipose tissue on the kidney rather than total body fat or body weight. [45]. Leptin induces mesangial hypertrophy in obese individuals [46]. In an experimental study, a decrease in adiponectin resulted in the fusion of podocyte foot processes and the development of CKD [47]. Most adipokines are known to be regulated by visceral adipose tissue rather than subcutaneous fat [48, 49]; thus WHR, which is an efficient indirect measurement method of visceral fat, can be a good indicator of CKD risk associated with “unhealthy” obesity and central adipose excess.

Another notable finding of our study is the difference between manually measured WHR and BIA-calculated WHR. Regarding our results, BIA-calculated WHR seems to be a better indicator of CKD risk than manually measured WHR in Cox regression and ROC analyses. The BIA measuring instrument used in this study is highly reliable, inexpensive, and uncomplicated for measuring muscle mass, body fat mass, lean mass, and water content. It has been verified and widely used to measure body fat percentages in clinical practice [5]. Measuring waist circumference and hip circumference with this instrument is based on the principle of calculating the volume of lean body mass for each part, and then calculating the circumference through the area [25]. According to previous studies, BIA-calculated waist circumference showed higher specificity and a lower false-positive rate for diagnosis of a visceral fat area > 100 cm^2^ on abdominal computed tomography compared to manually measured waist circumference [50–52]. WHR measured by BIA may be a more suitable metric to represent abdominal visceral fat, but further research is needed to evaluate the exact cause of this difference between manual and BIA-calculated body composition metrics.

This study not only verified the association of CKD development and obesity reported in previous observational studies, but also revealed that WHR measured by BIA was a better predictive indicator of CKD development than body weight, total fat mass, or total muscle mass, suggesting the unelucidated role of visceral obesity on the kidney. However, our study had several limitations. The etiology of CKD could not be assessed because of a lack of data. Direct measurement of the visceral fat area using image-based methods was not done. Computed tomography and MRI are known to be precise methods for calculating visceral fat volume, but high cost, accessibility, and the risk of radiation exposure are hurdles to its clinical application [53, 54]. However, as WHR and WC showed a high correlation with image-based visceral fat area in a previous study, WHR can be considered as an alternative to these image-based methods [55]. Another limitation of this study is that the WHR and fat content measuring instruments using the BIA method are outdated due to the long study period, raising questions about their current clinical application. However, our results provide evidence for the importance of measuring visceral fat rather than total body fat or mixed trunk fat (visceral and subcutaneous) for predicting renal dysfunction and can be the basis for further analysis using various novel BIA machines in the field of kidney research.

The substantially increasing incidence and prevalence of CKD worldwide is presumed to be caused by an increase in various underlying diseases such as diabetes and hypertension, but an increase in anthropological problems such as obesity, especially central obesity, is also estimated to be a significant cause [42]. According to our study findings, we suggest measuring WHR using the BIA method, rather than measuring BMI, to define obesity and to predict and manage the risk of CKD development. Further prospective studies are needed to determine a target range of visceral fat area for preserving kidney health, and to determine optimal strategies for managing visceral obesity to prevent kidney dysfunction.

## Data Availability

The data supporting the findings of this study are available in clinical database from Korean Genome and Epidemiology Study and are accessible after permission from http://is.cdc.go.kr (accessed on 1 July 2022).
